# Intraoperative Displacement of a Pterygoid Implant into the Pterygopalatine Fossa and Its Surgical Retrieval: A Case Report

**DOI:** 10.3390/dj14090592

**Published:** 2026-09-14

**Authors:** Horia Mihail Barbu, Andreea Sorina Petris, Stefania Andrada Iancu, Cosmin Ulman, Yarin Lorian Singer, Adi Lorean

**Affiliations:** 1 Oral Implantology Department, Faculty of Dental Medicine, Titu Maiorescu University, 031593 Bucharest, Romania; horia.barbu@gmail.com; 2 Prof. Dr. Barbu Academic Clinic, 011473 Bucharest, Romania; 3 Titu Maiorescu Doctoral School of Dental Medicine, 040441 Bucharest, Romania; 4 Department of Prosthodontics, Faculty of Dental Medicine, Titu Maiorescu University, 031593 Bucharest, Romania; 5 Trident Dental Clinic, 050533 Bucharest, Romania; cosmin.ulman@clinicatrident.ro; 6 Lorian Medical, Tel Aviv 6438123, Israel; yarinlorian2@gmail.com (Y.L.S.); adilorean@gmail.com (A.L.)

**Keywords:** pterygoid implants, full-arch rehabilitation, implant displacement, pterygopalatine fossa, cone-beam computed tomography

## Abstract

**Background/Objectives**: Pterygoid implants are increasingly used to provide distal support in full-arch maxillary rehabilitation, improving biomechanical stability and reducing posterior cantilevers. Despite high reported survival rates, their placement is technically demanding because of the complex anatomy of the pterygomaxillary region and the proximity of critical neurovascular structures. Implant displacement into adjacent deep anatomical spaces is a rare but potentially serious complication. The aim of this report is to describe the management of sequential intraoperative complications involving pterygoid implants—initial migration into the maxillary sinus followed by displacement into the pterygopalatine fossa—and to analyse the technical factors that contributed to them. **Case Description**: A 68-year-old woman underwent full-arch maxillary rehabilitation with eight implants, including bilateral pterygoid implants. Both pterygoid implants showed progressively decreasing primary stability and migrated into the maxillary sinus. During a salvage attempt, a newly inserted pterygoid implant deviated and was displaced beyond the pyramidal process into the pterygopalatine fossa together with the insertion driver. Cone-beam computed tomography (CBCT) localized the implant—lying medial to the lateral pterygoid plate, anterior to the pterygoid process—and guided a targeted retrieval based on careful blunt dissection, performed in an outpatient setting without general anesthesia or endoscopic assistance. A correctly positioned pterygoid implant was then placed. The anterior implants (insertion torque > 60 N·cm) were immediately loaded; at four months the right pterygoid implant was uncovered, and the left implant retained in the sinus was retrieved and replaced using a trans-sinus technique with elevation of the Schneiderian membrane. **Conclusions**: This case highlights the importance of accurate, consistent anatomical localization, complete osteotomy preparation, appropriate implant selection, and controlled, torque-monitored insertion during pterygoid implant placement. It also demonstrates that rare displacement of an implant into a deep anatomical space can be safely managed in an outpatient dental setting by experienced clinicians using a structured, image-guided approach.

## 1. Introduction

Full-arch rehabilitation of the edentulous or severely compromised maxilla requires reliable posterior support to ensure biomechanical stability, optimal load distribution, and long-term prosthetic success. Achieving adequate distal anchorage is particularly challenging in the posterior maxilla due to advanced bone resorption, poor bone quality, and complex regional anatomy [1]. Specific anchorage concepts have therefore been developed to support immediate or early loading protocols in complete maxillary rehabilitation [2].

Pterygoid implants have been introduced as a strategic solution that provides distal support by engaging the pyramidal process of the palatine bone and/or the pterygoid process of the sphenoid bone [3]. When properly positioned they create a stable posterior support zone, reduce cantilever length, and improve biomechanical conditions, with recent studies reporting high survival rates—particularly when CBCT-based planning and experienced surgical execution are employed [4,5,6].

Despite these favorable outcomes, placement remains technically demanding because of the anatomical complexity of the pterygomaxillary region and the proximity of critical neurovascular structures, including the maxillary artery, the pterygoid venous plexus, and branches of the trigeminal nerve [7,8]. Primary stability is typically obtained through engagement of the pyramidal process of the palatine bone, with or without additional anchorage in the pterygoid process of the sphenoid, depending on implant length, angulation, and individual anatomy. Loss of primary stability, deviation from the planned trajectory, or inadequate engagement of these structures may result in severe intraoperative complications, including uncontrolled migration into adjacent deep anatomical spaces [2,9,10]. Computer-aided planning and static or dynamic guided protocols have been proposed specifically to reduce trajectory errors during pterygoid and full-arch maxillary rehabilitation [11,12,13].

Implant displacement into adjacent anatomical spaces is a rare but potentially serious complication of posterior maxillary implant surgery [14]. While migration into the maxillary sinus is comparatively frequent, displacement into deeper regions—such as the pterygopalatine or infratemporal fossa—is uncommon and poses significant surgical challenges [9]. Precise and consistent anatomical localization is essential: an implant visualized lateral to the lateral pterygoid plate should be considered to lie within the infratemporal fossa, whereas an implant located medial or anterior to the pterygoid process, behind the posterior wall of the maxillary sinus and in proximity to the maxillary nerve (V2), the pterygopalatine ganglion, and the terminal maxillary artery, should be regarded as occupying the pterygopalatine fossa.

The purpose of this case report is to describe an intraoperative displacement of a pterygoid implant into the pterygopalatine fossa during full-arch maxillary rehabilitation, to outline the CBCT-guided surgical retrieval strategy, to analyse the technical factors that led to the complication, and to discuss measures aimed at minimizing the risk of similar events.

## 2. Case Presentation

**Figure 1:**
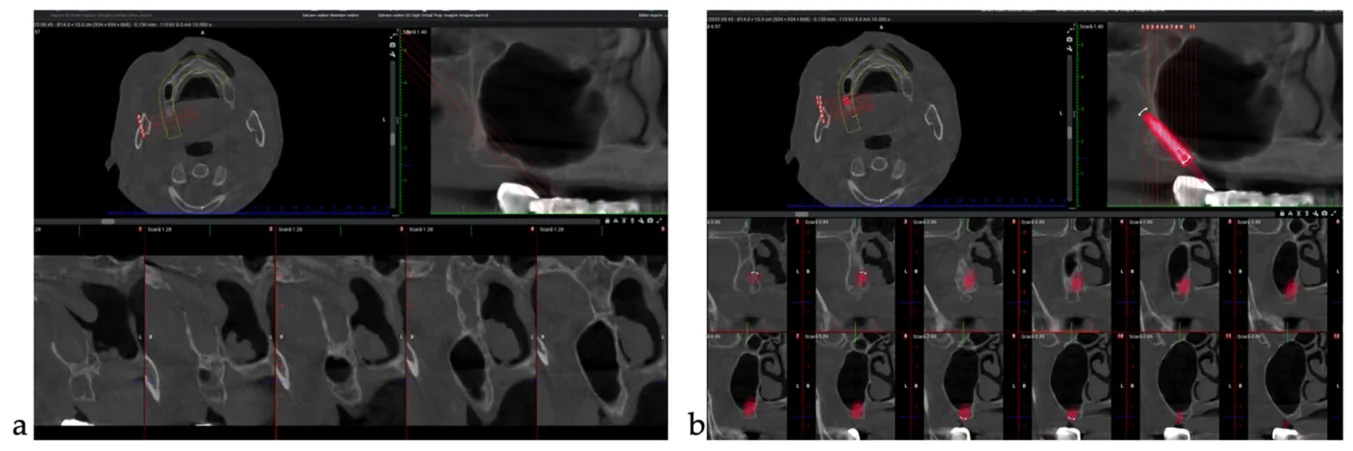
(**a**) Initial CBCT. (**b**) Pterygoid implant planning.

A 68-year-old female non-smoker with no relevant systemic comorbidities presented for full-arch maxillary rehabilitation with immediate loading. To avoid sinus floor augmentation, the treatment plan included bilateral posterior pterygoid implants for distal support (Figure 1). A full-thickness mucoperiosteal crestal incision was made from tuberosity to tuberosity; after flap elevation, the maxillary sinus, nasal floor and pterygoid region were identified.

### 2.1. Initial Surgery and Intraoperative Complications

In our routine protocol, pterygoid implants are placed from the maxillary tuberosity toward the pterygoid plates at an angulation of approximately 45° to the maxillary or Frankfort plane, engaging the pterygomaxillary junction while avoiding the sinus [3,7]. For the pterygoid sites in the present case, however, the operating surgeon selected a high, near-vertical approach, with the entry point at the third-molar position and an angulation of approximately 75–80° relative to the Frankfort horizontal plane, intended to reach the pterygoid plates through the maxillary tuberosity while avoiding the maxillary sinus, according to recent clinical and biomechanical data supporting steeper (around 70–80°) trajectories in the pterygomaxillary region [15,16,17]. The maxillary sinus was markedly pneumatized. Site preparation was performed using only 2.2 mm twist drills (Straumann), without a dedicated pterygoid osteotome sequence and without intraoperative confirmation (for example, with a depth probe) of whether the osteotomy had perforated the sinus floor.

**Figure 2:**
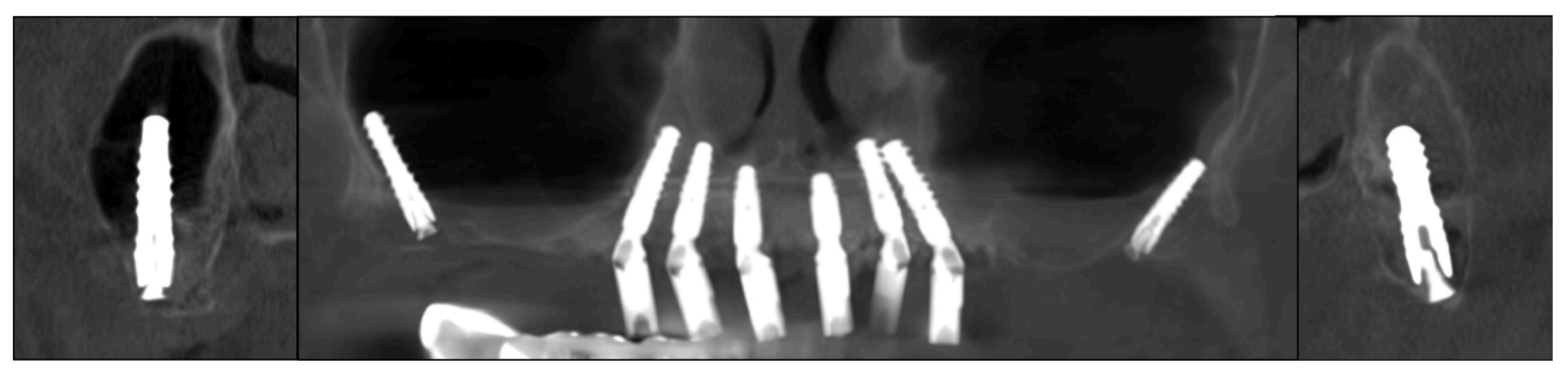
Postoperative CBCT showing displacement of both pterygoid implants into the maxillary sinus.

Two BLX implants (4.0 × 16 mm; Institut Straumann AG, Basel, Switzerland) were placed in positions 1.7 and 2.7. The BLX has a rounded, non-cutting apex; combined with an under-prepared osteotomy and the pneumatized sinus, no engagement of the pyramidal process was achieved. A progressive reduction in primary stability was observed during insertion (final insertion torque ≈ 15 N·cm), and the pterygoid implants were therefore excluded from immediate loading and cover screws were placed. While cover screws were being screwed in, the implants were seen to migrate progressively into the sinus cavity, with complete loss of primary stability. The six anterior implants, placed at the level of the nasal floor, achieved insertion torque above 60 N·cm. Postoperative CBCT confirmed displacement of both pterygoid implants into the maxillary sinus (Figure 2).

### 2.2. Salvage Attempt and Displacement into the Pterygopalatine Fossa

**Figure 3:**
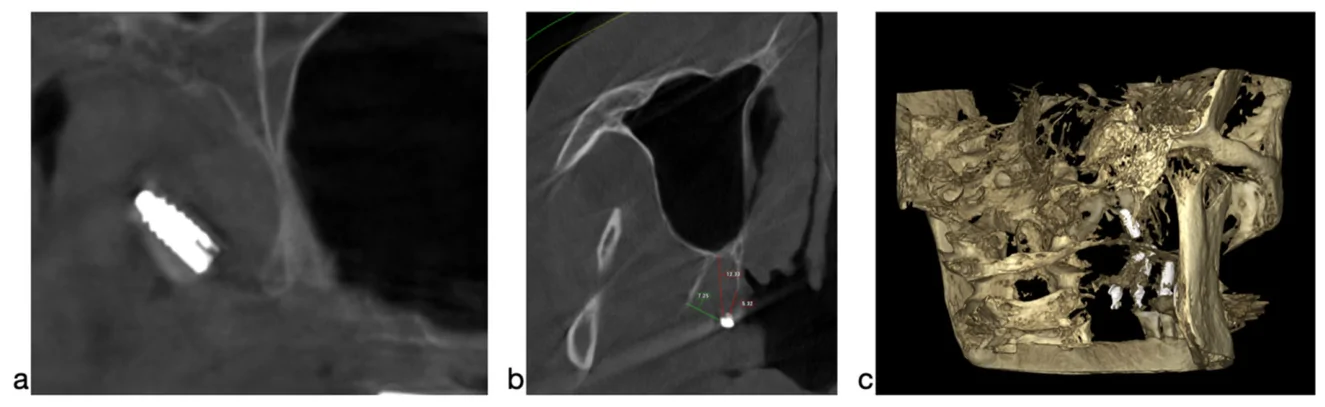
CBCT localization of the displaced implant. (**a**) Sagittal view showing the displacement. (**b**) Axial view showing the implant medial to the lateral pterygoid plate, anterior to the pterygoid process, within the pterygopalatine fossa. (**c**) Three-dimensional volume rendering of the displaced implant.

Immediate re-intervention was undertaken. The flap was reopened in quadrant 1 and the displaced sinus implant was retrieved. An attempt to place a new pterygoid implant was then made. Rather than re-planning the trajectory with CBCT, the existing socket was enlarged for access and the osteotomy was redirected more inferiorly through the same entry site using the 2.2 mm drill alone, leaving the osteotomy under-prepared. A regular BLX implant (3.75 × 10 mm—short because of the position at the third-molar site) was engaged in this neo-osteotomy and then advanced manually with a straight mount driver, without torque control or incremental verification. The implant then slipped beyond the pyramidal process and disappeared, together with the implant driver, into the pterygopalatine fossa; only a small portion of the driver remained visible intraorally. The driver was withdrawn by the surgeon, hoping to have the implant with it, but the highly engaging threads kept the implant fixed in the fossa. Postoperative CBCT confirmed migration of approximately 8 mm beyond the pyramidal process into the pterygopalatine fossa, the implant lying medial to the lateral pterygoid plate, anterior to the pterygoid process (Figure 3).

### 2.3. CBCT-Guided Retrieval and Correct Repositioning

**Figure 4:**
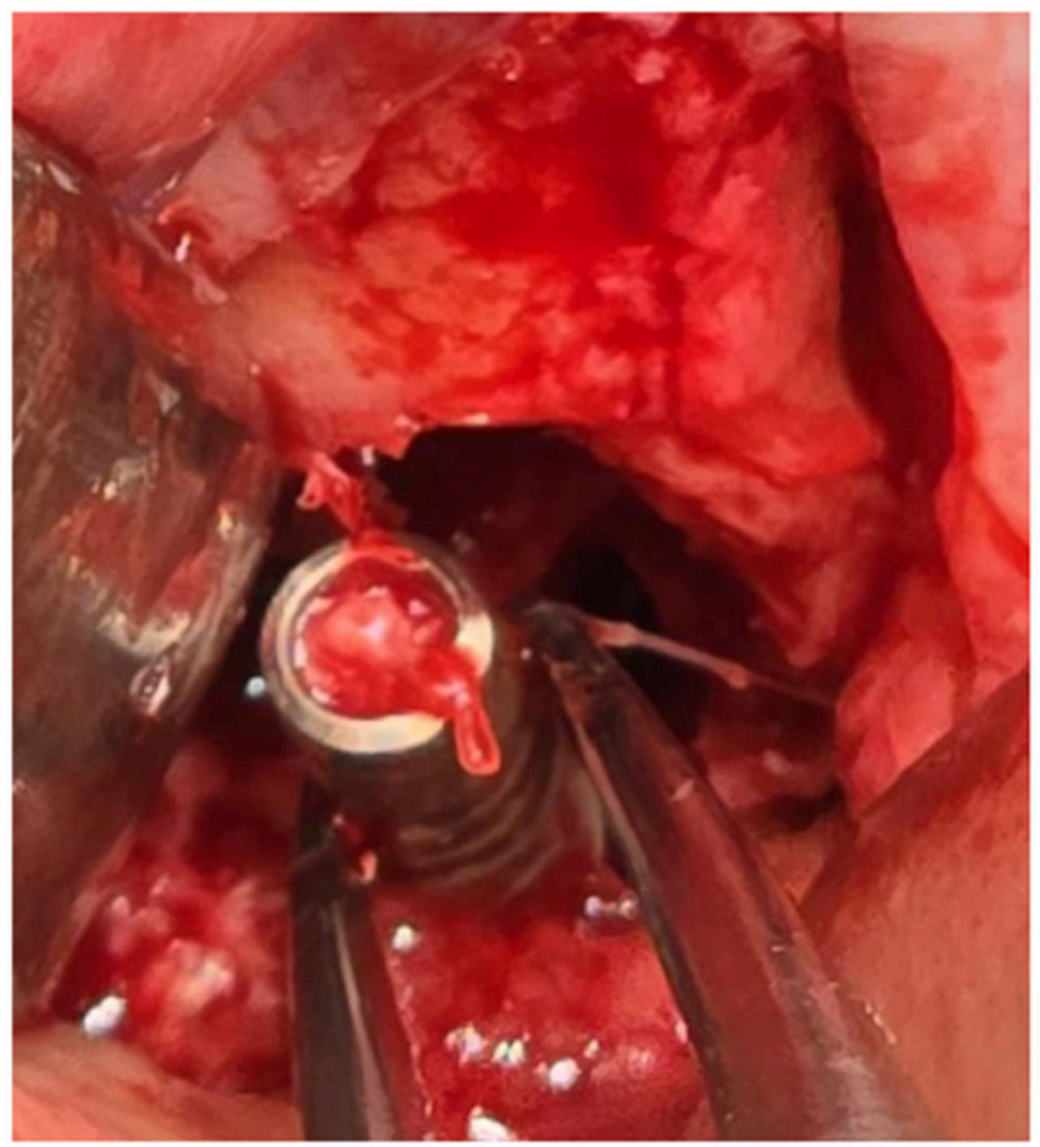
Clinical image of the displaced pterygoid implant after retrieval from the pterygopalatine fossa.

**Figure 5:**
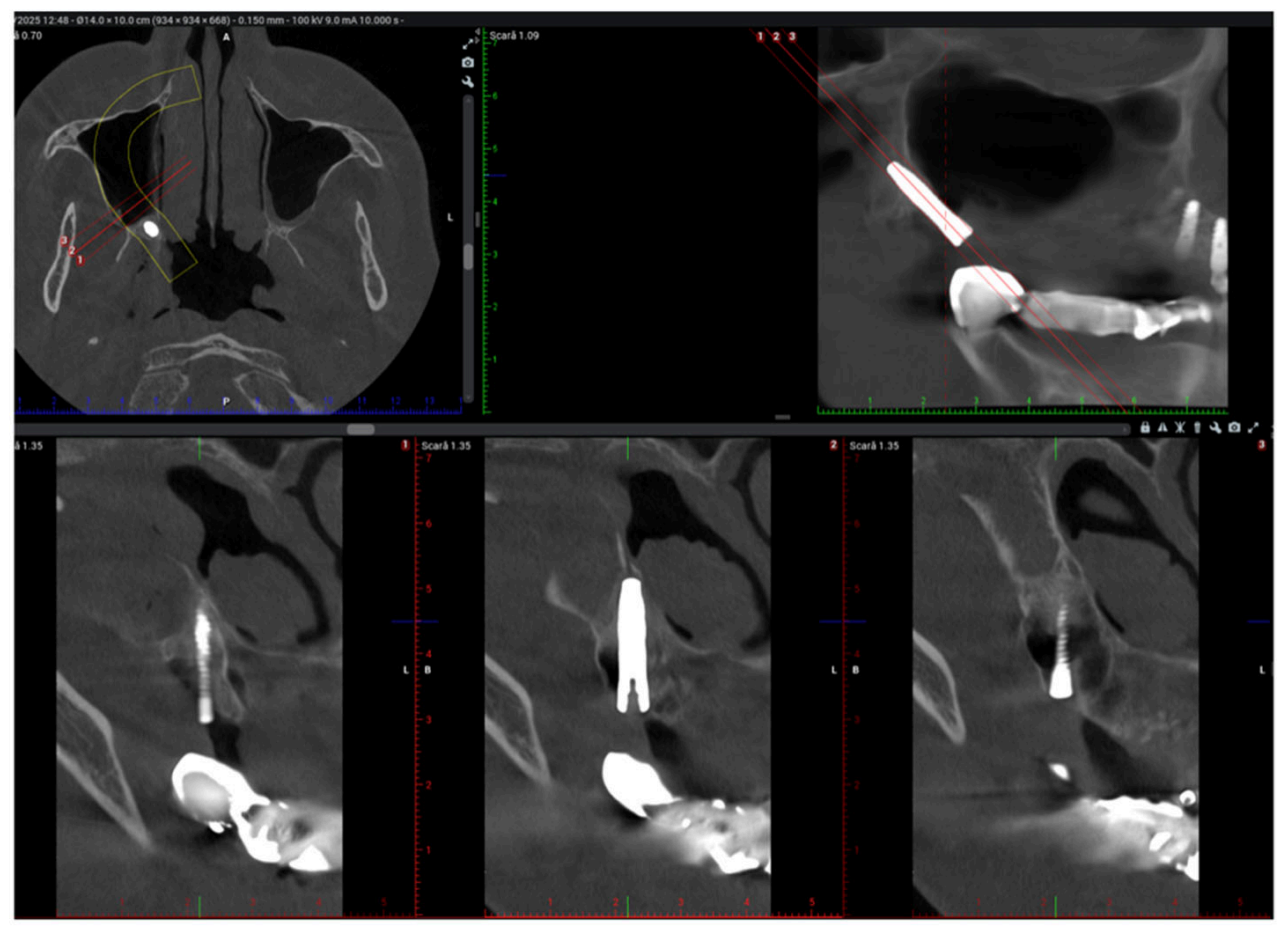
Immediate postoperative CBCT confirming proper positioning of the newly placed right pterygoid implant.

A second surgeon (the first author) was consulted and a targeted, CBCT-guided approach was planned. The distal incision was extended and careful blunt dissection was carried out toward the implant along the measured trajectory, following the direction aimed by the surgeon and the mount driver, from buccal to medial. Close contact with bone was maintained throughout in order to protect the contents of the pterygopalatine fossa—the maxillary nerve, the pterygopalatine ganglion and the terminal maxillary artery—and the adjacent pterygoid venous plexus. Because no standardized technique for retrieving an implant displaced into the pterygopalatine fossa has been described in the literature, we first attempted retrieval by extending the existing surgical access under CBCT guidance rather than resorting to endoscopy or a separate approach; the implant was located a short, CBCT-measurable distance along the same trajectory and was therefore within reach of this dissection, thereby avoiding the additional instrumentation and operating time of an endoscopic procedure. After approximately 15 min the implant was palpated with surgical forceps and removed without bleeding or injury to adjacent structures (Figure 4). A new osteotomy aiming directly at the medial pterygoid plate was performed using only the 2.2/2.8 and 3.5 drills for easier engagement of the tip of the 4.5 mm diameter TLC implant (Straumann), used as a pterygoid implant, which was placed on the right side according to the author’s technique (Section 3.1). A 4.5 × 18 mm Straumann TLC implant was inserted at 45° posterior angulation and 15° medial inclination, achieving 65–70 N·cm; given the complexity of the incident it was left to osseointegrate without immediate loading (Figure 5).

### 2.4. Follow-Up and Outcome

The six anterior implants, which had achieved insertion torque above 60 N·cm, were immediately loaded with the planned provisional fixed prosthesis; the pterygoid implants were excluded from immediate loading and managed in a staged fashion. At four months, second-stage surgery confirmed successful osseointegration of the right pterygoid implant (Straumann TLC 4.5 × 18 mm, position 1.7), which was uncovered. At the same session, the left pterygoid implant that had remained displaced in the maxillary sinus (position 2.7) was retrieved. First, the cover screw was removed and then, using a special driver from the Straumann surgical kit that screws directly onto the implant, the implant was removed.

**Figure 6:**
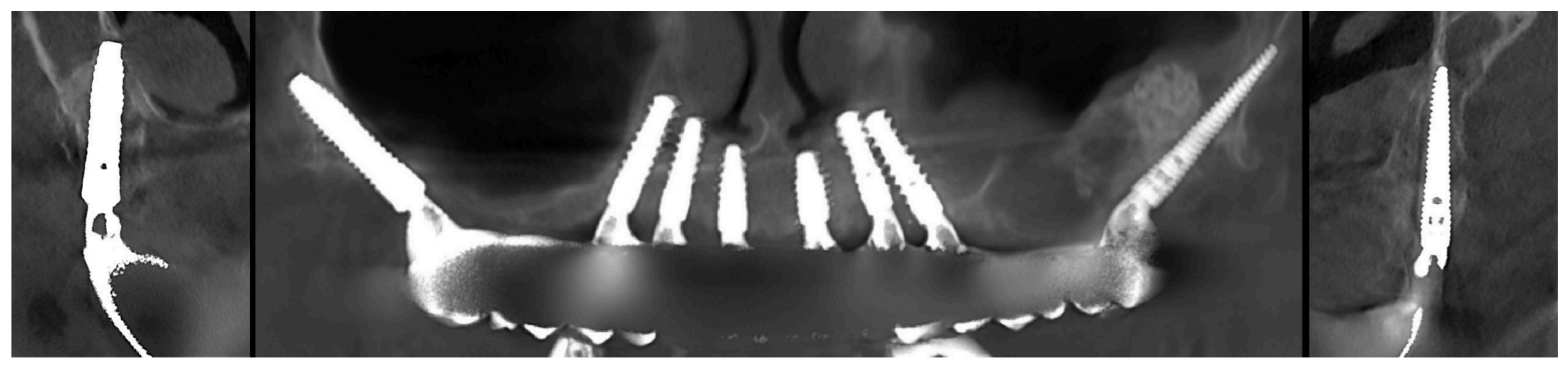
Follow-up CBCT, showing both definitive pterygoid implants correctly positioned and osseointegrated (right: Straumann TLC 4.5 × 18 mm; left: Southern Implants 4.0 × 24 mm, placed trans-sinus), with the anterior implants in function.

The perforated Schneiderian membrane was elevated and then sutured. Then, using the same osteotomy path, a new pterygoid implant was placed through a trans-sinus approach: the drills and then the implant were advanced under direct vision, and a new pterygoid implant (Southern Implants, Irene, South Africa; 4.0 × 24 mm) was placed. The bony defect left by the removed implant dictated the position of the new 24 mm length implant. A primary stability of 35 N·cm was obtained; xenograft was then added beneath and around the implant. A follow-up CBCT obtained four months after this second-stage surgery demonstrated both definitive pterygoid implants correctly positioned and osseointegrated, with the anterior implants in function (Figure 6).

**Figure 7:**
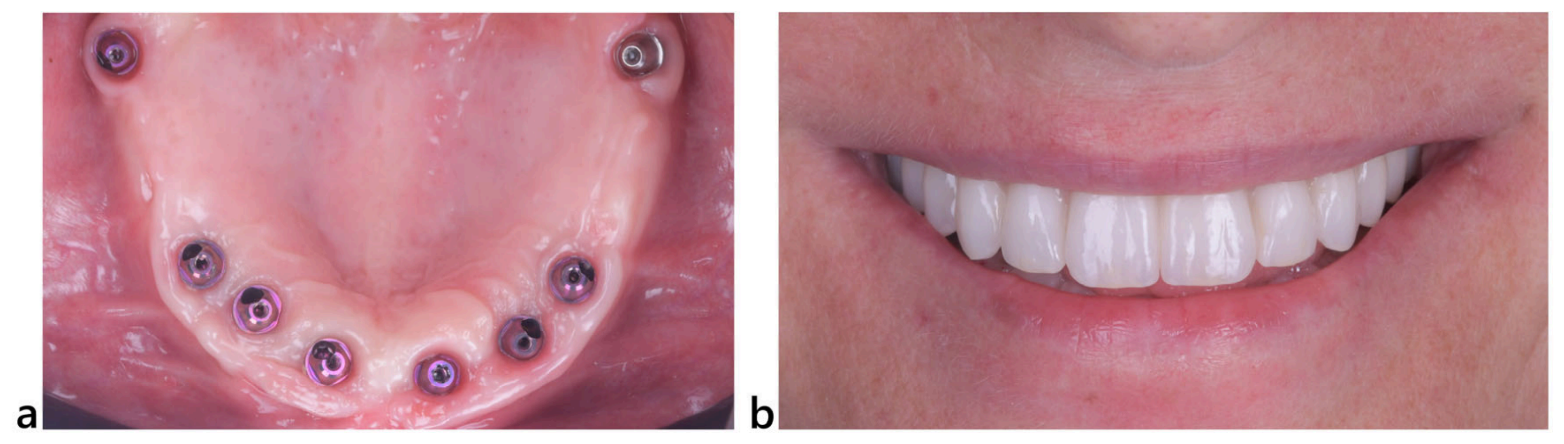
Intraoral view (**a**) of the prosthetic abutments and extraoral view (**b**) of the definitive implant-supported prosthesis made of titanium, zirconia, and ceramic.

Healing was uneventful throughout. The definitive titanium-fused-to-zirconia (Figure 7) fixed prosthesis was delivered four months after placement of the last pterygoid implant. At the most recent review—a total follow-up of nine months, including one month after delivery of the definitive restoration—both pterygoid implants and the prosthesis were stable and fully functional, with no neurological, vascular or sinus sequelae and a satisfactory patient-reported outcome.

## 3. Discussion

Pterygoid implants shorten treatment time by minimizing the need for sinus floor augmentation, and the combination of local bone density, regional anatomy and implant design supports immediate loading protocols, with reported success rates of 88–99% [4,5,6,9,18]. Prosthetically, the pterygoid implant acts as the distal pillar of the restoration, eliminating the occlusal stress of a distal cantilever [1,2]; compared with conventional All-on-4 protocols [19], an additional pterygoid implant improves occlusal load distribution and protects the peri-implant bone [20].

The principal disadvantage is the surgical difficulty arising from the anatomical complexity of the region, which requires an experienced surgeon [7]. The intended site is difficult to access and has limited direct visibility [21]; inter-arch space is restricted, and the implant neck and implant–abutment connection at the second-molar level can complicate wide opening during surgery and the prosthetic phase [22]. The region is rich in important neurovascular structures [8], and correct angulation and depth demand careful preoperative CBCT planning [7,11].

### 3.1. Two Surgical Approaches and the Author’s Preferred Technique

Two surgical approaches to the pterygomaxillary region are described, differing in entry point and angulation and selected largely according to sinus pneumatization and tuberosity dimensions [3,7].

In the vertical, posterior approach, the implant enters at about the third-molar position with a steep, near-vertical angulation and traverses the maxillary tuberosity and pyramidal process to engage the pterygoid plates, while aiming to avoid the maxillary sinus. It suits limited sinus pneumatization with adequate tuberosity bone but is performed largely blind and has limited indications when the sinus is pneumatized.

In the oblique, anterior approach, which the author prefers, the implant enters at the second-molar position with an approximately 45° antero-posterior and 15–25° bucco-palatal angulation, starting about 14 mm from the lateral pterygoid plate, and is directed through the pyramidal process to anchor in the pterygoid plates—consistent with the classic Tulasne trajectory [3] and with reported angulation ranges [7].

Trans-sinus variant for the pneumatized sinus. When the sinus is markedly pneumatized—as in the present case—the author does not apply a blind trans-sinus trajectory. A posterior access is created in the lateral sinus wall and the Schneiderian membrane is elevated; the osteotomy is prepared 14 mm anterior to the pyramidal process so that the drill passes through the sinus under direct vision, enters the posterior sinus wall and then the pyramidal process, aiming to anchor in the medial pterygoid plate. Autogenous and/or bovine bone is then grafted beneath and around the implant. A blind trans-antral maneuver should be avoided, being difficult even for surgeons with extensive pterygoid experience. Usually, these pterygoid-trans-sinus implants are not immediately loaded.

*Site preparation.* The author uses sequential osteotome preparation (Noris Pterygoid Surgical Set; Noris Medical, Haifa, Israel) with biological drilling at reduced speed (≤50 rpm) without irrigation, both to harvest autogenous bone and to condense the cancellous bone [23], confirming firm resistance at the pyramidal process before insertion, followed by controlled, incremental, torque-monitored seating.

*Implant selection and length rationale.* To engage the cortical bone between the pyramidal and pterygoid processes at 45° from a point 14 mm anterior to the lateral plate, an implant of a mean length of 20 mm is generally required (range 18–22 mm). Straumann implants are used routinely in the author’s clinic, and the 18 mm TLC has been recently adopted as the preferred implant for the pterygoid site, its additional 1.8 mm machined (polished) collar giving a length of approximately 20 mm.

In this medial trajectory, the buccal aspect of the implant neck frequently becomes extra-osseous and lies in permanent contact with oral mucosa; in such situations, a machined transmucosal collar is generally considered more favorable for plaque control and soft-tissue tolerance than an exposed roughened surface [24]. The TLC also has a sharper, more apically engaging tip than the BLX and achieves a better primary stability in this area.

### 3.2. Critical Analysis of the Contributing Factors

Looking back on this case, the complication resulted from a sequence of three errors rather than a single event. First, a high, near-vertical third-molar trajectory was chosen to avoid the pneumatized sinus but was executed without intraoperative confirmation of sinus integrity; combined with the rounded, non-cutting apex of the BLX implant and an under-dimensioned osteotomy, no engagement of the pyramidal process was obtained and the implant slid into the sinus—recognized only as the cover screw was being seated. Second, the salvage attempt was made without CBCT re-planning: the socket was enlarged and the trajectory redirected inferiorly through the same site with the 2.2 mm drill alone, producing an under-prepared osteotomy, in the hope that the implant tip would engage and provide primary stability sufficient for osseointegration. Third, a BLX implant poorly suited to actively engage dense cortical bone was forcefully driven manually with a straight mount driver, without torque control or incremental verification; because the thick, rounded, non-self-cutting tip of the implant did not engage in the 2.2 mm osteotomy, it slipped and was displaced beyond the pyramidal process into the pterygopalatine fossa together with the driver.

Taken together, these observations—drawn directly from the present case—support a clear principle: adequate rotary and osteotome preparation along the full intended path, an apically engaging implant, intraoperative confirmation of sinus integrity, and a controlled, torque-monitored insertion are essential to prevent accidents like uncontrolled migration in pterygoid implant surgery.

### 3.3. Localization, Retrieval and Preventive Considerations

**Figure 8:**
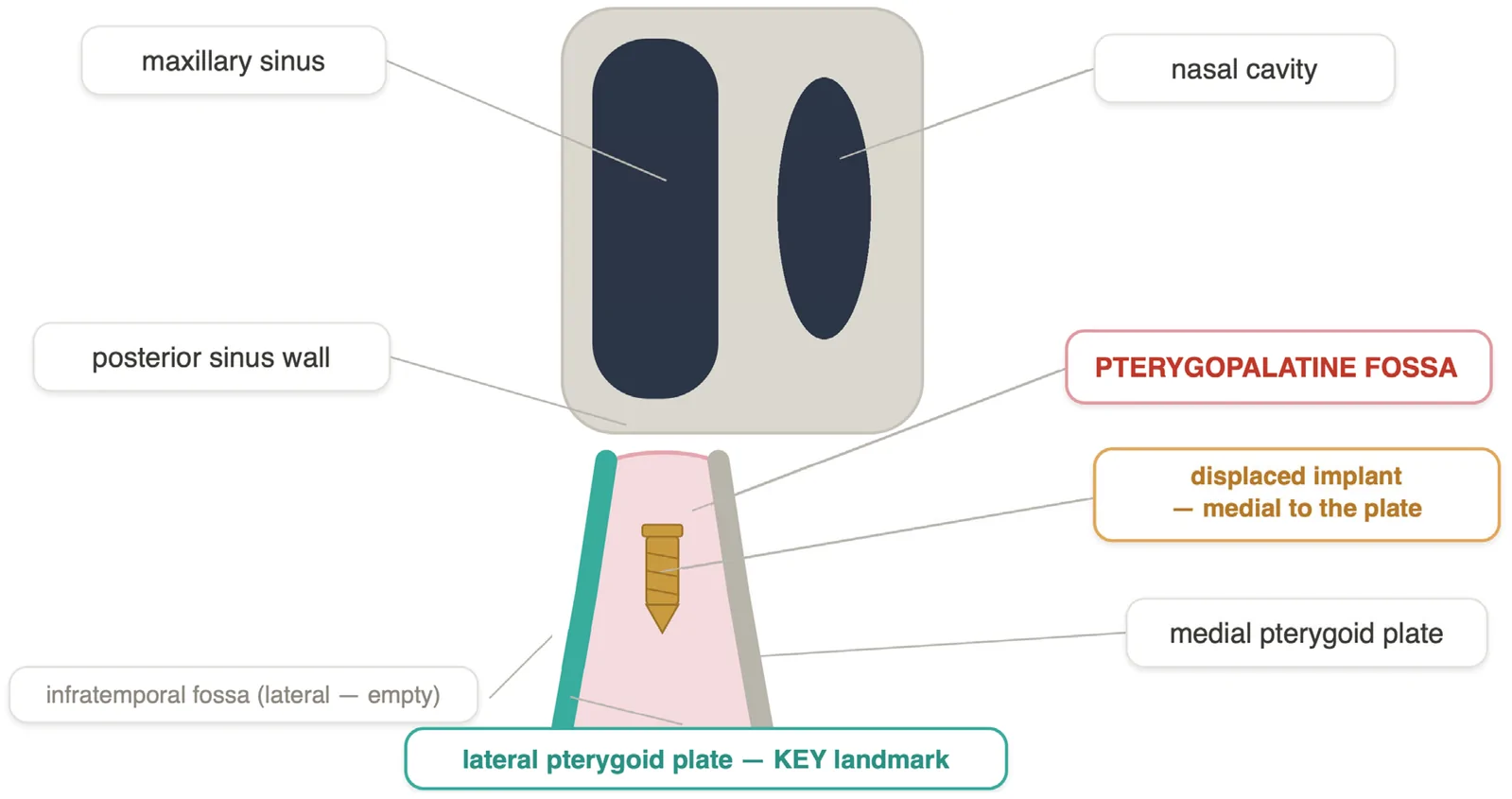
Schematic of the displaced-implant location.

Implant displacement during insertion or delayed migration most often involves the maxillary sinus because of its proximity to the posterior implant site [4,14]. Less commonly, implants are displaced into the nasal cavity, the sublingual or submental spaces, the pterygopalatine/infratemporal region, or the orbit [25,26,27,28,29]. Because the present case began with migration into the maxillary sinus before the subsequent displacement, the literature on prompt diagnosis, appropriate planning, and timely retrieval of sinus-displaced implants is directly relevant [14,26]. The pterygopalatine fossa contains critical neurovascular structures—including the maxillary nerve (V2), the pterygopalatine ganglion and the terminal maxillary artery, with the pterygoid venous plexus immediately adjacent—so uncontrolled migration into this space carries risks of bleeding, pain, and nerve injury [30]; in this case, early recognition, immediate CBCT, and a measurement-guided, stepwise blunt dissection allowed safe removal without neurovascular injury, underscoring the value of three-dimensional imaging both for planning and for complication management. The anatomical basis for distinguishing the pterygopalatine fossa from the infratemporal fossa, and the position of the displaced implant relative to the lateral pterygoid plate, are summarized schematically in Figure 8.

To place the sinus component of this case in a broader context, a 2023 systematic review of 321 cases of dental implants displaced into the maxillary sinus reported that displacement occurred within the first six months in 62.6% of cases, that 56.2% of patients became symptomatic (mainly maxillary sinusitis and/or oroantral communication), and that surgical removal was ultimately required in 93% of cases, most often via a transoral lateral or Caldwell–Luc approach (65%) or transnasal endoscopy (23%) [31]. These figures support the strategy adopted here—prompt recognition and planned, staged removal rather than watchful observation—once the displaced implant was confirmed radiographically.

Approach selection for sinus-displaced implants is increasingly individualized according to implant location, ostium patency, and the presence of sinusitis or an oroantral communication. A comparative analysis of four transoral, non-endoscopic approaches found that operative time and morbidity rose sharply with the depth and posterior extent of access—from a mean of 11.5 min for implants retrievable through the existing alveolar/crestal site to 42 min for implants requiring a posterior lateral window, the latter also carrying a higher risk of bleeding from the pterygoid venous plexus [32]. Where sinus outflow is compromised or sinusitis is present, transnasal endoscopic sinus surgery has been reported to safely restore ostial patency while avoiding an external bony window, and is increasingly favored as the first-line approach in symptomatic cases [33,34]. Conversely, a direct transoral or Caldwell–Luc approach remains appropriate when the implant is readily accessible through the existing surgical site or when concurrent oroantral fistula repair is required [35]. In the present case, the sinus-displaced implant was retrieved immediately through the existing osteotomy—analogous to the low-morbidity alveolar approach described above—without need for a lateral window or endoscopic access, consistent with its anterior, readily accessible position. This individualized selection of approach, guided by implant location rather than a single standardized technique, mirrors the treatment algorithms now emerging from the sinus-retrieval literature. The subsequent, more complex event reported here—secondary displacement into the pterygopalatine fossa during the salvage procedure—falls outside this better-characterized sinus-retrieval paradigm and, to our knowledge, has not been previously described in comparable detail, underscoring the primary contribution of the present case.

The lateral pterygoid plate is the key landmark: the implant lies medial to it, anterior to the pterygoid process, in the pterygopalatine fossa behind the posterior wall of the maxillary sinus and adjacent to the maxillary nerve, the pterygopalatine ganglion and the terminal maxillary artery; the infratemporal fossa lateral to the plate is empty.

Several measures may reduce the risk of similar events. Careful preoperative CBCT planning—including evaluation of the pterygoid process, maxillary tuberosity and sinus—is essential to determine angulation, length and position, since bone quality and morphology directly affect placement accuracy [11,30,36]. For complex cases, a patient-specific three-dimensional (printed or digital) model derived from the initial CBCT can improve 3D understanding of the relationship between the tuberosity, pyramidal process and pterygoid plates, and allows the surgeon to measure and verify angulation and to rehearse placement; this should be regarded as a useful tool in selected complex cases rather than a routine standard of care, as the present case cannot prove that its absence caused the complication or that its use would have prevented it. Static and dynamic computer-aided protocols have likewise been proposed to reduce trajectory errors in pterygoid and full-arch maxillary rehabilitation [12,13].

In addition, use of a dedicated pterygoid surgical kit with the full drilling/osteotome sequence and an apically engaging, pterygoid-appropriate implant reduces displacement and migration. Mount drivers incorporating mechanical implant-retention features may reduce the risk of accidental fixture detachment and posterior uncontrolled advancement during insertion in the distal maxilla. Finally, when resistance, loss of tactile control, unexpected depth, or suspected sinus involvement is encountered, intraoperative verification of the trajectory—with a depth probe and/or CBCT—should be mandatory before the implant is inserted.

### 3.4. Practical Recommendations

**Figure 9:**
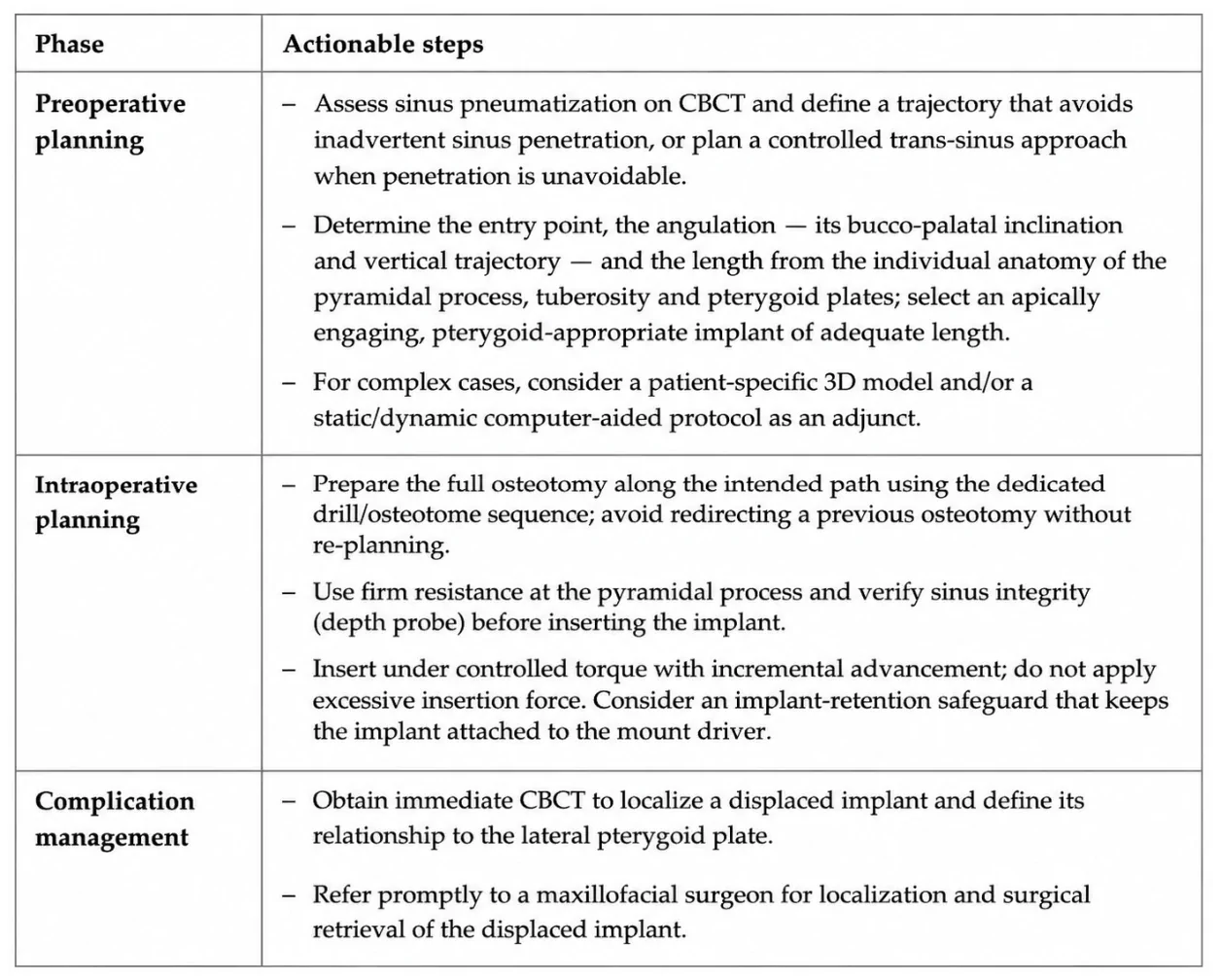
Phase-based clinical recommendations for pterygoid implant placement and for the early management of intraoperative implant displacement Phase-based clinical recommendations for pterygoid implant placement and for the early management of intraoperative implant displacement.

On the basis of the present case and the reviewed literature, we propose a phase-based set of clinical recommendations to reduce the risk of implant displacement during pterygoid implant placement and to support the early recognition and management of this complication (Figure 9).

## 4. Conclusions

Pterygoid implants are a valuable option for full-arch maxillary rehabilitation, but their placement demands precise anatomical knowledge, appropriate instrumentation and implant selection, and a controlled surgical technique. This case demonstrates that a blind trajectory through a pneumatized sinus, an under-prepared osteotomy, an implant geometry inappropriate for this site, and uncontrolled manual force can lead to implant migration into the sinus or displacement into the pterygopalatine fossa. Surgeons should rely on thorough CBCT-based planning, complete drilling/osteotome sequences, intraoperative verification of the trajectory and sinus integrity, and careful, torque-monitored insertion, and should be prepared to manage rare but serious complications through targeted, image-guided surgical retrieval. Patient-specific 3D models and computer-aided protocols may be useful adjuncts in selected complex cases.

## Data Availability

The data presented in this study are available on request from the corresponding authors due to privacy reasons.
